# Effects of ectoine containing nasal spray and eye drops on symptoms of seasonal allergic rhinoconjunctivitis

**DOI:** 10.1002/clt2.12006

**Published:** 2021-03-24

**Authors:** Anne M. Salapatek, Nina Werkhäuser, Basma Ismail, Ralph Mösges, Esther Raskopf, Andreas Bilstein

**Affiliations:** ^1^ Cliantha Research Mississauga Ontario Canada; ^2^ Bitop AG Witten Germany; ^3^ CRI—Clinical Research International Ltd. Cologne Germany; ^4^ ClinCompetence Cologne GmbH Cologne Germany

**Keywords:** ARC, ectoine, EEC, environmental exposure chamber, seasonal allergic rhinoconjunctivitis, total nasal symptom score, total ocular symptom score, Allergische Rhinoconjunctivitis, Ectoin, Pollenkammer, Gesamt‐Nasen‐Symptom‐Score, Gesamt‐Augen‐Symptom‐Score

## Abstract

**Background:**

Patients are often dissatisfied with the symptom control obtained from available pharmacological treatments for seasonal allergic rhinoconjunctivitis (ARC). Therefore, patients seek for alternative, nonpharmacological options to treat their symptoms. Here, we assessed the efficacy of ectoine nasal spray and ectoine eye drops in comparison to placebo to prevent nasal and ocular symptoms following exposure to pollen in patients with ARC.

**Methods:**

In this double‐blind, randomized, placebo‐controlled, cross‐over study, 46 patients with ARC applied ectoine eye drops and nasal spray in immediate succession or placebo eye drops and nasal spray for 13 days before ARC symptoms were induced in an environmental exposure chamber. Primary endpoint was the baseline‐adjusted area under the curve (AUC) posttreatment total nasal symptom score (TNSS) and the total ocular symptom score (TOSS) using analysis of covariance. Secondary endpoints were, amongst others, total nonnasal symptoms score (TNNSS) and nasal patency (measured using acoustic rhinometry).

**Results:**

Treatment with both ectoine and placebo reduced TNSS, TOSS, and TNNSS upon allergen exposure. The analysis of parameters at baseline and after allergen exposure demonstrated that ectoine induced a clinically relevant improvement in ARC symptoms compared to placebo: the least square mean difference for baseline‐adjusted AUC was ‒1.87 for TNSS, ‒1.45 for TOSS and ‒2.20 for TNNSS. The mean change from baseline AUC of TNNSS for ectoine was also significantly greater than for placebo (‒5.49 vs. ‒3.46; *p* = 0.011). Ectoine significantly improved the singular symptoms “sneezing,” “watery eyes” and “itchy eyes” (*p* ≤ 0.021) as well as “itchy ear/palate” (*p* = 0.036) in comparison to placebo. Mean cross sectional areas of the nasal cavity were reduced to a lesser extent after treatment with ectoine (‒0.020 ± 0.022) than with placebo (‒0.047 ± 0.029). The current study also demonstrated a very good safety profile of ectoine treatment. Few AEs with comparable numbers in both treatment groups were reported during the study, which were mild in severity and resolved without medical treatment.

**Conclusion:**

The study suggests that ectoine is effective in reducing nasal and ocular symptoms associated with ARC. Being a natural, bacteria derived stress protection molecule functioning by a physical mode of action, it therefore represents an alternative nonpharmacological treatment option.

## 1 BACKGROUND

Despite the vast number of drug‐based treatments available for seasonal allergic rhinoconjunctivitis (ARC),^1^ patient dissatisfaction is still an issue. This is partially due to the available medications which do not control the signs and symptoms adequately enough to match the patients' needs,^2^, ^3^, ^4^, ^5^ or because the drugs can induce deleterious side effects.^6^, ^7^, ^8^, ^9^

Thus, there is medical need for alternative treatment options. Most of these alternatives are nonpharmacological treatment options used by the patients as shown in previous literature.^10^, ^11^, ^12^, ^13^

The extremolyte ectoine is one of these prospective, alternative, nonpharmacological treatment options. It is an osmolyte of low molecular weight which is generated by extremophilic bacteria and protects biological molecules from external influences such as extreme temperature, pressure, salt concentration, and ultraviolet radiation. Ectoine protects macromolecules (like membranes and proteins) via an entropy‐driven, cosmotropic physical mechanism.^14^, ^15^ This leads to the stabilization of the native form of proteins, as well as an increase in the fluidity of lipid membranes,^16^, ^17^ thus resulting in the increased stability of the membrane barrier to various stressors.^18^, ^19^ Ectoine reduces the inflammatory process at the membrane level, as was shown in different preclinical models.^20^, ^21^, ^22^ Ectoine is currently used in topical applications for skin diseases such as atopic dermatitis.^20^, ^23^ Ectoine nasal spray (ENS) has also been studied in patients with acute rhinosinusitis.^24^ To evaluate the general efficacy and safety of ENS for treating allergic rhinitis or rhinoconjunctivitis, several clinical trials have assessed it compared to antihistamines, cromoglycate, and to glucocorticoids. Thus, in a comparative, open‐label study,^25^ a nasal spray containing ectoine was as effective as cromoglycate for relieving rhinoconjunctivitis symptoms and—because of virtually no side effects—was tolerated significantly better by the patients. Additionally, the ENS used in conjunction with ophthalmic drops containing ectoine were as effective as the corresponding azelastine products in relieving allergic symptoms during the pollen season.^25^ In an open randomized study with children, the use of nasal ectoine in combination with oral antihistamines reduced significantly faster the severity of the rhinitis symptoms nasal congestion, nasal discharge, nasal irritation, and sneezing, as well as conjunctivitis symptoms like itchy eyes and conjunctival hyperemia in comparison to oral antihistamines alone.^26^

However, the objective measurement of allergy‐related symptoms remains a challenge. To overcome this, the present study used a validated environmental exposure chamber (EEC) model which is capable of examining anti‐allergic treatments by mimicking a natural, yet controlled airborne allergen exposure. Thus, confounding variables such as unpredictable atmospheric pollen levels due to erratic weather conditions with climate changes seen in traditional field trials can be eliminated.

In this proof‐of‐concept study, we aimed to assess the extent of relief of rhinoconjunctivitis symptoms through cotreatment with both nasal and ocular applications of ectoine compared to placebo.

## 2 METHODS

### 2.1 Approvals and ethics

The study protocol and informed consent form were approved by Institutional Review Board Services (ON, Canada), and written informed consent was obtained from all patients prior to enrollment in the study. The study was conducted in accordance with good clinical practice, the ethical principles of the Declaration of Helsinki (Seoul 2008) and Health Canada's regulations and guidelines. The trial registration was from ClinicalTrials.gov Identifier: NCT01471184, Date of registration: November 15 2011 (retrospectively registered); URL: https://clinicaltrials.gov/ct2/show/NCT01471184?term=NCT01471184&draw=2&rank=1.

### 2.2 Study design

**FIGURE 1:**
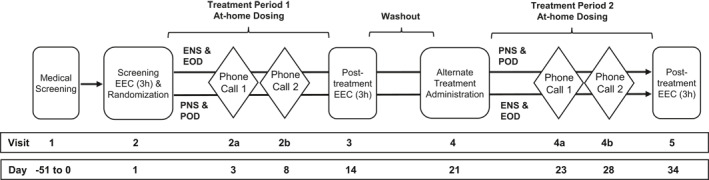
Treatment schedule/study conduct. The study comprised two screening visits (one medical and one environmental exposure chamber screening, EEC). Treatment was started at Visit 2. The patients continued the treatment for 13 days at home with two reminder phone calls in between (Visit 2a, 2b), before returning for a posttreatment EEC visit (Visit 3). Then, patients had a 7 days washout period and were crossed over to the alternate treatment at Visit 4. Similar to Period 1, treatment was continued for another 13 days. Then, a final EEC visit was performed (Visit 5, study end after 34 days). EEC, environmental exposure chamber

This was a single center, double‐blind, placebo‐controlled, two‐way crossover study, which was conducted outside of the ragweed pollen season (Fall‐Winter 2009–2010 in Mississauga, ON, Canada). The study consisted of two prophylactic treatment periods with a washout period in between. Adherence to treatment was documented on a daily basis by the patients. In total, five visits and four accompanying phone calls between the visits were conducted (Figure 1). Visit 1 (V1) was a screening visit to evaluate eligibility of the patients. At Visit 2 (V2), a second screening (based on the symptom scores after exposure to the allergen in the EEC) was done and treatment was started. The patients continued the treatment for 13 days at home with two reminder phone calls in between (V2a and V2b), before returning for a posttreatment EEC visit (V3). Following this, patients had a 7 days washout period and were crossed over to the alternate treatment at Visit 4 (V4). Similar to Period 1, treatment was continued for another 13 days. Then, a final EEC visit was performed (V5, study end).

### 2.3 Study patients

Patients aged 18–65 years with a clinical history of ARC for ragweed and a positive skin prick test to ragweed were included. Patients who met the eligibility criteria after the screening EEC visit (minimum 6 of 12 points for total nasal symptom score [TNSS], 2 of 3 points for the congestion score and 4 points of 9 points for total ocular symptom score [TOSS]) were allocated to one of the two treatment sequence groups. Usage of ocular/topical/oral/nasal antiallergic medication including antihistamines, anticholinergics, or cromolyn for at least 7 days and intranasal/inhaled or systemic corticosteroids or antidepressants for at least 14 days before study start and throughout the study was prohibited.

### 2.4 Study assessments

Patients underwent a 3‐h stay in an EEC with exposure to 3500 ± 500 ragweed pollen grains/m^3^. Rhinoconjunctivitis symptoms were assessed before and after entry to the EEC. In addition, symptoms were also evaluated every 30 min during the EEC sessions. The scores for the symptoms of runny nose, itchy nose, nasal congestion, and sneezing were summed to obtain the TNSS. The TOSS was the sum of itchy, red, and watery eye symptoms. The total nonnasal symptom score (TNNSS), comprising the ocular symptoms from the TOSS as well as itchy ear/palate was also collected. For grading of all symptoms, a 4‐point scale was used with 0 = none, 1 = mild, 2 = definite awareness of sign/symptom that is bothersome but tolerable and 3 = sign/symptom is hard to tolerate.

In addition, acoustic rhinometry (AcR) tests were performed to assess nasal patency before and after each EEC session (pre‐EEC and post‐EEC, respectively) and the mean cross‐sectional nasal areas (MCAs) were determined. For further details on AcR and MCA procedure, please refer to File S1. AcR was carried out by trained operators. Measurements were performed in triplicate, and—where possible—the same equipment and operator were used for the entire study duration for each patient.

### 2.5 Study treatments and timing

The ectoine eye drops (EOD, marketed name: Ectoin^®^ Allergy eye drops; Bitop AG) consisted of an isotonic aqueous solution comprising ectoine (2%), hydroxyethyl cellulose (approximately 0.78%, adjusted to a viscosity of 15 mm^2^/s), sodium chloride (0.44%), sodium dihydrogen phosphate dihydrate (0.0048%), sodium hydrogen phosphate dihydrate (0.0203%), and water. The total weight of one drop was 30 mg (containing 0.6 mg ectoine). The ENS (Ectoin^®^ Allergy nasal spray; Bitop AG) was a slightly hypertonic aqueous solution (approximately 420 mosmol/kg) comprising ectoine (2%), sodium chloride (0.9%), and water. The total weight of one puff was 140 mg (containing 2.8 mg ectoine).

The placebo ophthalmic drops (PODs) included the following ingredients: sodium chloride (0.9%), hydroxyethyl cellulose (approximately 0.63%, adjusted to a viscosity of 10 mm^2^/s), sodium dihydrogen phosphate dihydrate (0.0048%), sodium hydrogen phosphate dihydrate (0.0203%), and water. The placebo nasal spray (PNS) was a solution of sodium chloride (0.9%) and water. The eye drops and nasal sprays were identical regarding packaging/labelling and quality. All study products were packed in systems which are especially designed for the application of preservative free formulations. Thus, the eye drops were manufactured in the COMOD^®^ system (Ursapharm Technology), and the nasal sprays were manufactured in the 3K^®^ system (Ursatec Technology). Manufacturing was carried out at Ursapharm Arzneimittel GmbH in accordance with EN ISO 13485 and good manufacturing practice requirements. The products had to be applied consecutively in accordance with the instructions for use (four times daily: in the morning, at lunch time, in the evening and at bedtime).

### 2.6 Study objectives and endpoints

The primary objective was the assessment of the efficacy of ENS and EOD in comparison to PNS and POD, by evaluating TNSS and TOSS, with the primary endpoint being the baseline adjusted AUC of subject‐rated instantaneous posttreatment TNSS and TOSS. Secondary objectives were the evaluation of the TNNSS and individual nasal symptoms. The corresponding secondary endpoints were baseline‐adjusted AUC of subject‐rated instantaneous posttreatment TNNSS and single symptom scores (nasal congestion, red/watery/itchy eyes). Effects of the treatment on nasal patency using the MCA were assessed with AcR by analyzing the change from baseline. An additional secondary endpoint was the evaluation of the safety of treatments, as assessed by the occurrence of adverse events (AEs).

### 2.7 Statistical analysis

Statistical analyses were performed using SPSS version 22 (IBM Corp). Baseline for all parameters was defined as the area under the curve (AUC) at V2. Change from baseline in mean (+standard error of the mean [+SEM]) posttreatment scores for TNSS, TOSS, and TNNSS was assessed for both active treatment and placebo. As primary analysis, mean (+95% confidence interval [CI]) AUC was assessed for active treatments compared to placebo using an analysis of covariance with baseline data as covariates. The least square means for each treatment and the least square mean differences (LSMDs) were calculated. A paired *t*‐test was performed by treatment to address changes from baseline in AUC for both TNSS and TOSS, as well as for TNNSS. A treatment difference that reached *p* < 0.05 was considered statistically significant, and a treatment difference of at least one score point on TNSS/TOSS/TNNSS was considered clinically meaningful (defined as the minimal clinically important difference^27^, ^28^). Posttreatment MCA change from pre‐EEC to post‐EEC at V3 and V5 was expressed as change from baseline values. Baseline was defined as the MCA value change from pre‐EEC to post‐EEC at V2. Descriptive statistics were used for baseline, posttreatment (V3 and V5) and change from baseline scores. Safety analysis was done by summarizing AEs by incidence (number and frequency) and treatment. Graphical images were prepared with the GraphPad Prism 8 (GraphPad Software).

## 3 RESULTS

### 3.1 Demographic data and baseline values

**FIGURE 2:**
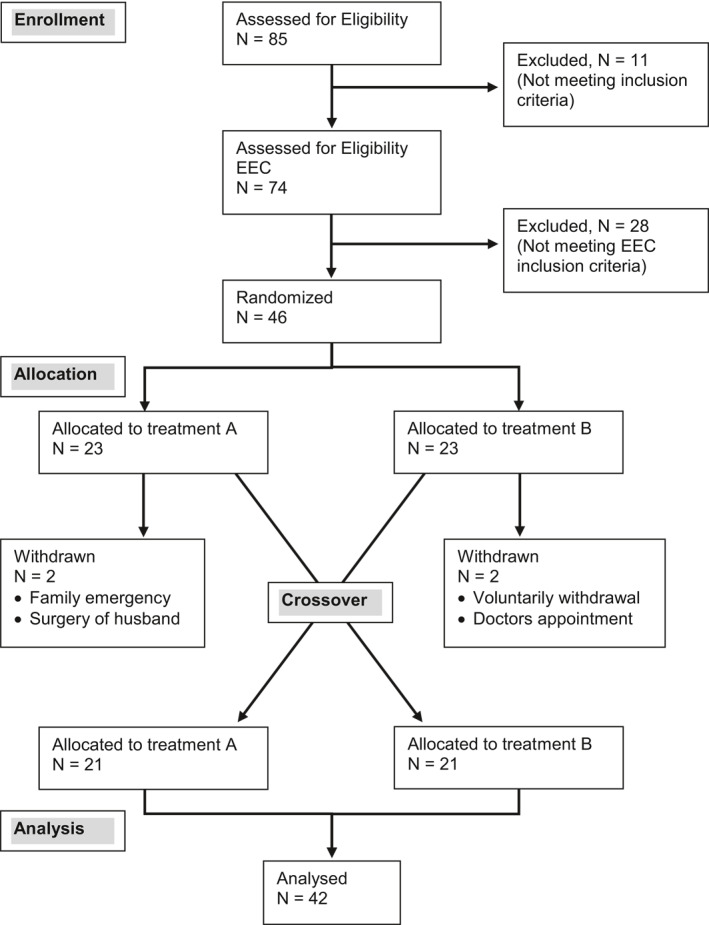
CONSORT Flow diagram of the study population

Of the 85 patients screened at V1, 46 patients were randomized to one of the two treatment sequences (ENS + EOD then PNS + POD or vice versa) after EEC at V2. For CONSORT Flow diagram of the study, please refer to Figure 2. Four patients (8.7%, two from the ectoine and two from the placebo group) withdrew from the study during the first treatment period. In accordance with the clinical study protocol definition of analysis sets, those patients were excluded from the intent‐to‐treat (ITT) population as they did not participate in any posttreatment efficacy assessment. No patient was excluded because of nonadherence to the treatment. In total, 42 patients completed all posttreatment assessments and had no major protocol deviations. They were constituted to the per protocol (PP) population, which was identical to the ITT population.

Patients' demography and age distribution are shown in Table 1.

**TABLE 1:** Summary of demographic and baseline characteristics

Parameters	ENS/EOD +	PNS/POD +	Total (*N* = 46)
	PNS/POD	ENS/EOD	
	(*N* = 23)	(*N* = 23)	
Origin	*n* (%)	*n* (%)	*n* (%)
American Indian/Alaska native	2 (8.7%)	0 (0%)	2 (4.3%)
Asian	1 (4.4%)	4 (17.4%)	5 (10.9%)
Black or African American	8 (34.8%)	6 (26.1%)	14 (30.4%)
Native Hawaiian or other Pacific Islander	1 (4.4%)	0 (0%)	1 (2.2%)
White	10 (43.5%)	11 (47.8%)	21 (45.7%)
Mixed^a^	1 (4.4%)	2 (8.7%)	3 (6.5%)
Ethnicity	*n* (%)	*n* (%)	*n* (%)
Hispanic or Latino	5 (21.7%)	1 (4.4%)	6 (13.0%)
Not Hispanic or Latino	18 (78.3%)	22 (95.6%)	40 (87.0%)
Gender	*n* (%)	*n* (%)	*n* (%)
Male	12 (52.2%)	10 (43.5%)	22 (47.8%)
Female	11 (47.8%)	13 (56.5%)	24 (52.2%)
Age	Years	Years	Years
Mean ± SD	44.0 ± 8.78	43.8 ± 13.61	43.9 ± 11.3
Min, max	30, 60	22, 65	22, 65

*Note*: Shown are the data of all randomized patients.

Abbreviations: %,  percentage based on *N*; ENS/EOD, treatment with ectoine nasal spray and eye drops; *N*, number of subjects randomized; *n*, number of subjects with data available; PNS/POD, treatment with placebo nasal spray and eye drops; SD, standard deviation.

^a^ Subjects who checked two or more races were classified as mixed.

### 3.2 Effect of ectoine on nasal symptoms (TNSS)

**FIGURE 3:**
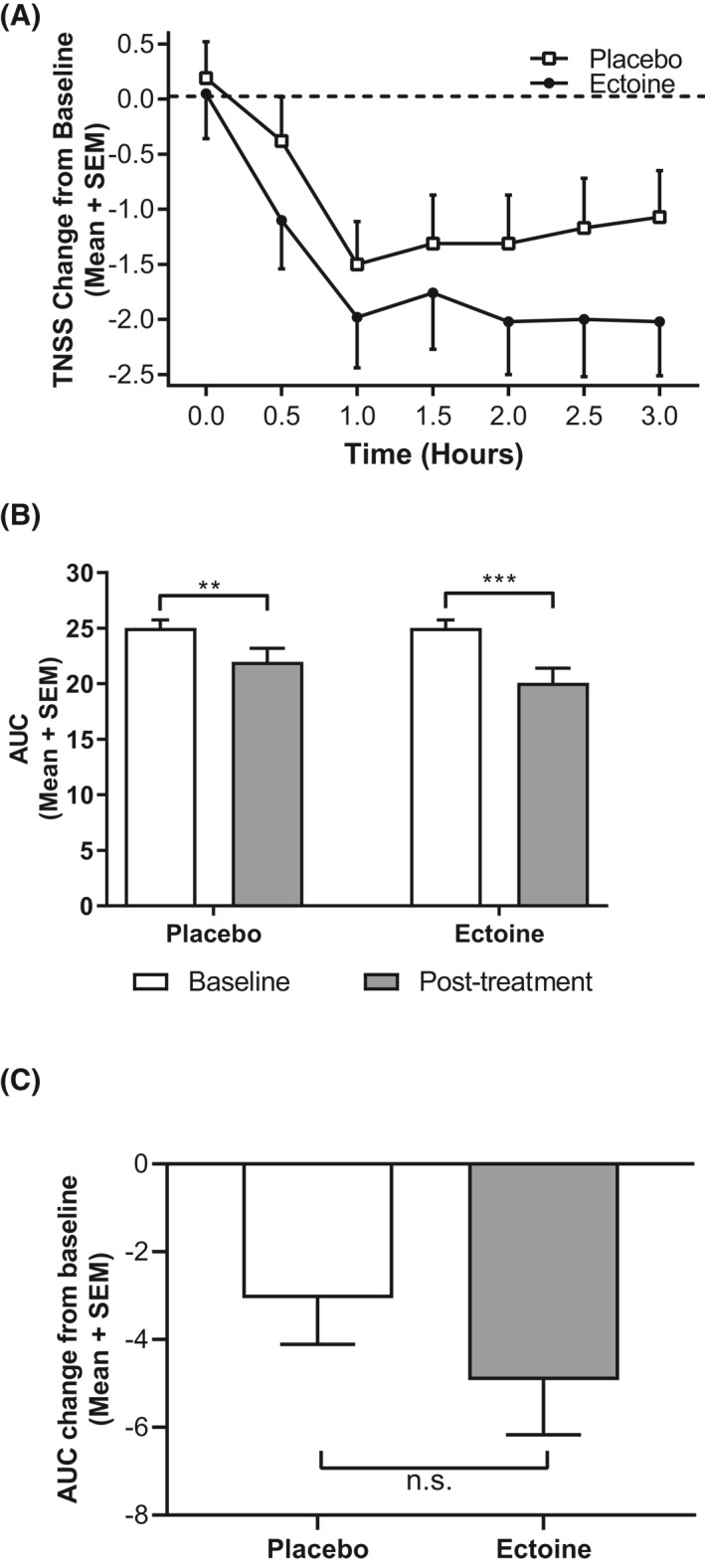
Total nasal symptom score (TNSS). Data are expressed as mean + SEM. (A) TNSS change from baseline revealed larger decreases for patients receiving ectoine at every time point in the EEC. Baseline was defined as the symptom scores at V2. (B) Mean TNSS area under the curve (AUC) scores showed a reduction under ectoine and placebo treatment (***p = 0.0003, **p = 0.006) comparing pretreatment baseline values at V2 and posttreatment values (V3 and V5). (C) Patients receiving ectoine experienced larger reductions in TNSS than placebo‐treated patients as shown by AUC change from baseline. EEC, environmental exposure chamber; SEM, standard error of the mean

Patients receiving ENS and EOD had a 1.89‐fold lower TNSS after 3 h of posttreatment EEC exposures than patients treated with placebo, though the TNSS decreased not only after ectoine, but also after placebo treatment when compared to baseline (Figure 3A). The mean (+SEM) AUC TNSS score was 25.02 ± 0.722 at the EEC screening visit, which was significantly reduced to 20.10 ± 1.31 (‒19.7%; *p* = 0.0003) following ectoine treatment at the posttreatment EEC visits. This was diminished too in patients who were treated with placebo (by 12.2%, 21.96 ± 1.21; *p* = 0.006; Figure 3B). This corresponds to a 7.47% greater reduction at the posttreatment visit by the ectoine treatment than in placebo‐treated patients. The mean reduction from baseline AUC of TNSS was 1.61‐fold greater in patients treated with ectoine when compared to placebo (LSMD: ‒4.92 vs. ‒3.05; Figure 3C, Table 2). This difference showed clinically meaningful improvement in the ectoine treatment group in comparison to placebo, but values did not reach statistical significance (*p* = 0.065).

**TABLE 2:** ANCOVA analysis of change from baseline AUC values (least squares mean difference, LSMD)

	Difference of ectoine versus placebo		
Parameter	LSMD	95% CI^a^	*p* ^a^
TNSS	‒1.869	‒3.860, 0.122	0.0651
Sneezing	‒0.762	‒1.377, ‒0.147	0.0198*
Itchy nose	‒0.607	‒1.238, 0.024	0.0666
Runny nose	0.393	‒0.179, 0.965	0.1861
Nasal congestion	‒0.286	‒0.766, 0.195	0.2364
TOSS	‒1.446	‒2.679, ‒0.213	0.0227*
Watery eye	‒0.577	‒1.057, ‒0.098	0.0195*
Itchy eye	‒0.595	‒1.096, ‒0.095	0.0210*
Red eye	‒0.274	‒0.733, 0.186	0.2355
TNNSS	‒2.024	‒3.552, ‒0.495	0.0108*
Itchy ear/palate	‒0.619	‒1.234, ‒0.004	0.0356*

*Note:* Statistical analysis of symptoms comparing ectoine versus placebo in a two‐period cross‐over design. Each row presents the mean difference between ectoine and placebo with its 95% confidence interval and p‐value (N = 42). Negative values of the treatment differences indicate reduction in symptoms after applying ectoine in comparison to placebo.

Abbreviations: 95% CI, 95% confidence interval; ANCOVA, analysis of covariance; AUC, area under the curve; TNSS, total nasal symptom score; TNNSS, total nonnasal symptom score; TOSS, total ocular symptom score.

^a^ 95% Confidence intervals and *p*‐values are based on the linear mixed model for a crossover design, where the outcome variable is the adjusted AUC by baseline values, treatment and period are considered as fixed effects and subject nested within sequence as a random effect.

*Statistically significant.

### 3.3 Effect of ectoine on ocular symptoms (TOSS)

**FIGURE 4:**
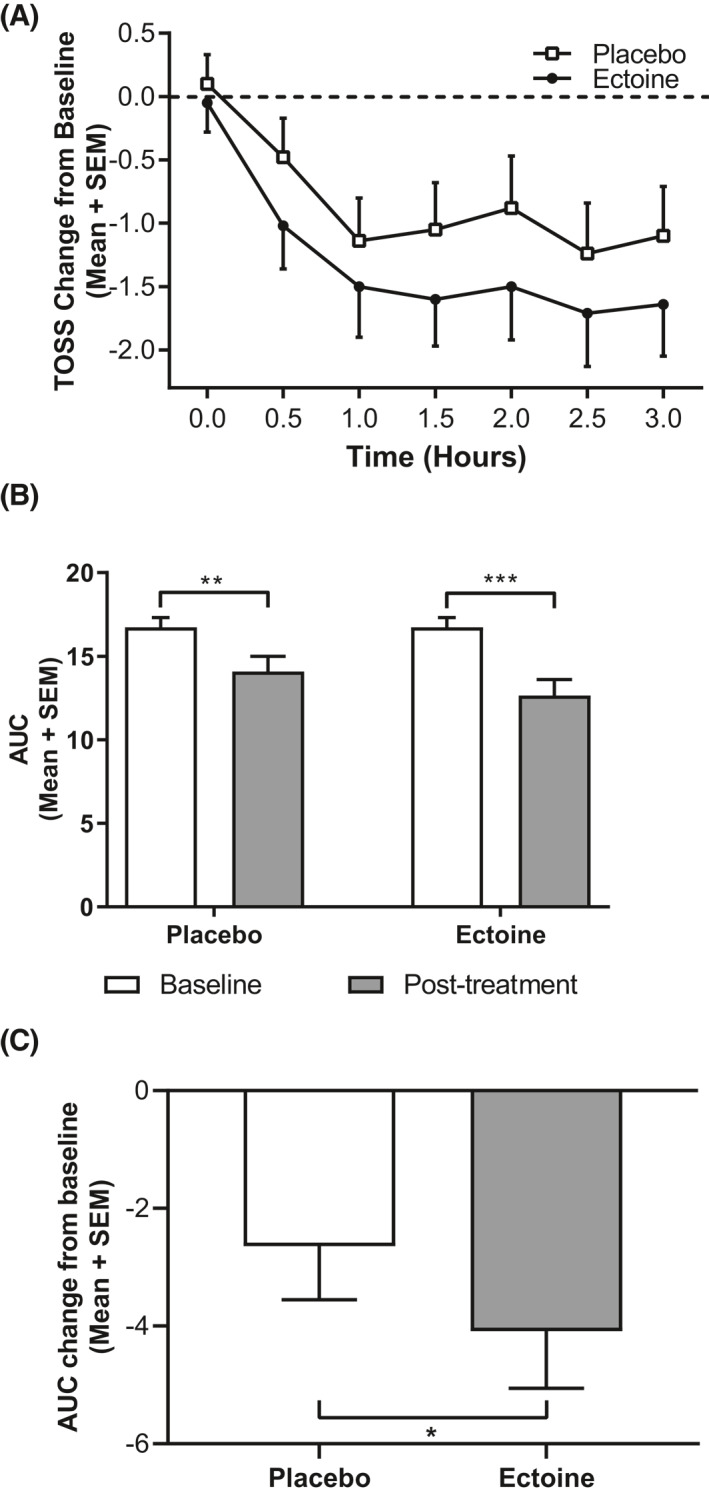
Total ocular symptom score (TOSS). Data are expressed as mean + SEM. (A) TOSS change from baseline exhibited larger decreases for patients receiving ectoine at every time point in the EEC. Baseline is defined as the symptom scores at V2. (B) Mean TOSS area under the curve (AUC ) scores showed a significant reduction with ectoine and placebo treatment (***p = 0.0001, **p = 0.0059) comparing baseline values at V2 and posttreatment values (V3 and V5). (C) Patients receiving ectoine experienced larger reductions in TOSS than placebo‐treated patients as shown by AUC change from baseline. This reduction was statistically significant compared to placebo treatment (*p = 0.0227). EEC, environmental exposure chamber; SEM, standard error of the mean

Similarly, to the effects on nasal symptoms, both treatments reduced ocular symptoms in comparison to the screening EEC. Overall patients receiving ENS/EOD experienced a mean 1.49‐fold greater relief of overall ocular symptoms scores after 3 h of posttreatment EEC when compared to PNS/POD (Figure 4A). The mean AUC for TOSS at the EEC screening visit was 16.73 ± 0.58. At the posttreatment EECs, TOSS significantly decreased to 12.64 ± 0.97 (‒24.4%; *p* = 0.0001) after ectoine treatment and to 14.09 ± 0.91 (‒15.8%; *p* = 0.0059) after treatment with placebo (Figure 4B). This corresponded to an 8.6% greater reduction from baseline to posttreatment EEC in ectoine‐treated patients in comparison to placebo. The results also revealed a greater decrease (1.55‐fold) in the mean change from baseline AUC of TOSS for ectoine (LSMD: ‒4.09) compared to placebo (LSMD: ‒2.64; *p* = 0.023; Figure 4C, Table 2). This difference showed both clinically meaningful and statistically significant improvement in the TOSS after ectoine treatment in comparison to placebo treatment.

### 3.4 Effect of ectoine on nonnasal symptoms (TNNSS)

**FIGURE 5:**
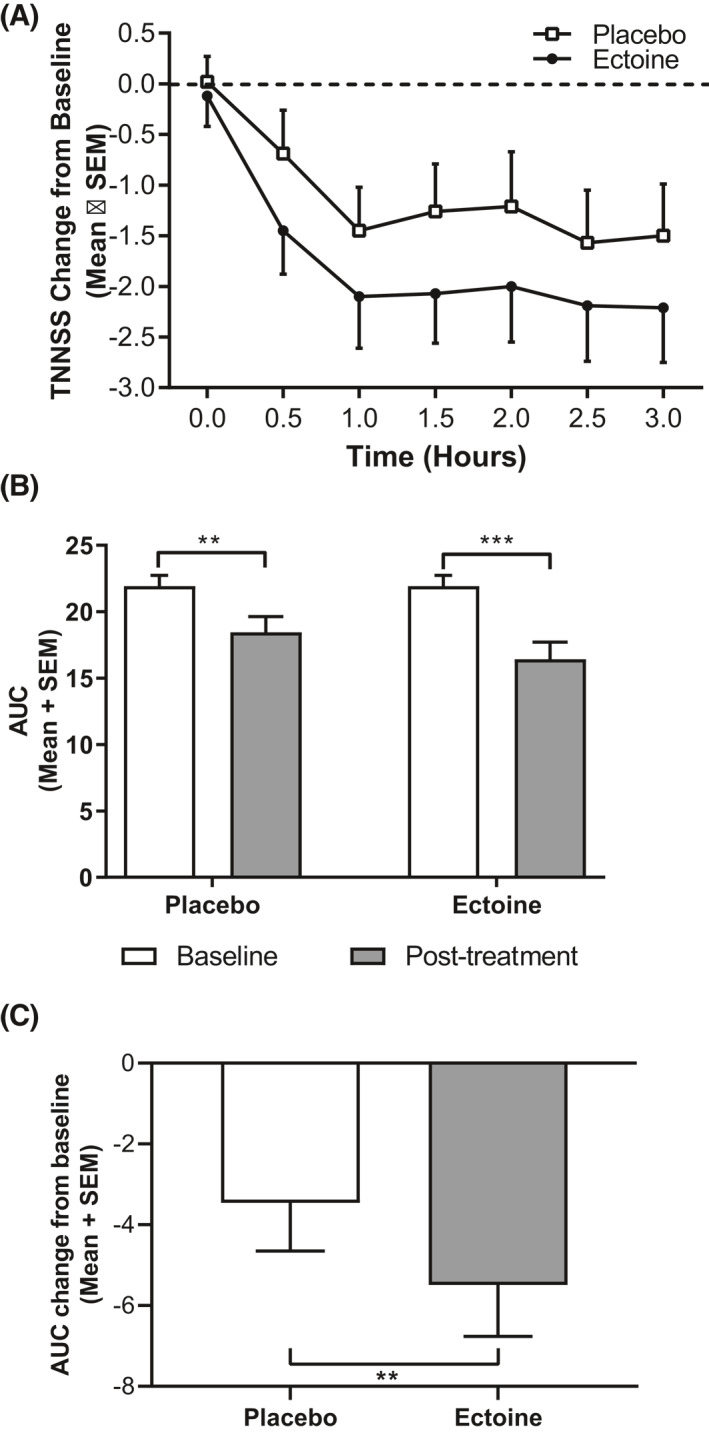
Total non‐nasal symptom score (TNNSS). Data are expressed as mean + SEM. (A) TNNSS change from baseline revealed larger decreases for patients receiving ectoine at every time point in the EEC. Baseline is defined as the symptom scores at Visit 2. (B) Mean TNNSS area under the curve (AUC) scores showed a significant reduction under ectoine and placebo treatment (***p < 0.0001, **p = 0.0059). (C) Patients receiving ectoine experienced statistically significant reductions in TNNSS when compared to placebo, as shown by the percent change from baseline (**p = 0.0108). EEC, environmental exposure chamber; SEM, standard error of the mean

The TNNSS also showed a larger decrease in the baseline adjusted EEC (1.47‐fold after 3‐h exposure) in patients treated with ectoine than in patients treated with placebo, although the TNNSS was also reduced in the placebo‐treated patients when compared to baseline (Figure 5A). The mean AUC of the TNNSS was 21.92 ± 0.822 at the EEC screening visit. This decreased to 16.44 ± 1.271 (‒25.03%; *p* > 0.0001) and to 18.46 ± 1.18 (‒15.80%; *p* = 0.0059) in patients treated with ectoine and placebo, respectively, resulting in a significantly greater reduction (by 9.23%; *p* < 0.0001) in the ectoine‐treated patients (Figure 5B, Table 2). The mean reduction from baseline AUC of TNNSS for ectoine (‒5.49) was significantly greater (1.59‐fold; *p* = 0.011) compared to placebo (‒3.46; Figure 5C).

### 3.5 Effect of ectoine on single symptoms

When investigating individual nasal symptoms, treatment with ectoine resulted in significantly greater relief of the symptom “sneezing” (*p* = 0.020; Table 2), whereas the differences in the changes in the other nasal symptoms were not significant.

For individual ocular symptoms, mean change from baseline AUC values were more reduced after ectoine treatment than with placebo, with a statistically significant greater relief for “watery eyes” (*p* = 0.020) and “itchy eyes” (*p* = 0.021): The baseline AUC for “watery eyes” was 5.70 at the EEC screening visit, which decreased to 4.80 (‒15.77%) in placebo‐treated patients and to 4.23 (‒25.89%) in patients who were treated with ectoine. Similarly, the baseline AUC LSMD for “itchy eyes” was 5.94 at the EEC screening visit, which decreased to 4.98 (‒16.23%) and 4.38 (‒26.26%) at the posttreatment EEC following placebo ectoine treatment, respectively (Table 2).

The itchy ear/palate symptom of the TNNSS had a treatment LSMD of 0.62, which was also statistically significant (*p* = 0.036; Table 2).

### 3.6 Acoustic rhinometry

Analysis of the objective measure of change in nasal patency showed that ectoine treatment reduced the MCA to a lesser extent (‒0.020 ± 0.022) than placebo (‒0.047 ± 0.029), but without statistical significance (*p* = 0.29). The least square mean for the MCA change was also smaller in the ectoine group than in the placebo group (‒0.018 ± 0.021 vs. ‒0.049 ± 0.021) with a LSMD of 0.031 (*p* = 0.298).

### 3.7 Safety

Five (11.4%) of the forty‐four patients who administered ENS/EOD reported six AEs. No patient withdrew from the study because of an AE. One AE, an upper respiratory tract infection, was of moderate severity and was not considered as related to the treatment. All other AEs reported in the study were rated as mild. One patient reported burning eyes which was considered unrelated to treatment by the investigator. Of the three AEs possibly related to treatment were anxiety, headache and mild conjunctivitis, all of which resolved without medical treatment within 24 h. Two patients (4.5%) who administered placebo reported five AEs: one patient experienced fatigue, drowsiness and back pain, while the other reported mild headache and nasal discomfort. These AEs were rated as mild and not related to the treatment.

## 4 DISCUSSION

This study aimed to investigate whether ectoine could alter the natural development of rhinoconjunctivitis signs and symptoms in patients exposed to airborne allergens. We were able to show that ectoine treatment effectively reduced the hallmark symptoms of rhinoconjunctivitis with minimal side effects. Symptoms relief was clinically meaningful in the primary endpoint analyses of TNSS and TOSS and showed a statistically significant benefit over placebo for TOSS.

It is widely accepted that the prevalence of ARC is increasing all over the world. Treatment options have been thoroughly investigated in many studies which resulted in the development of guidelines for an optimal approach.^1^, ^29^, ^30^ According to these guidelines, oral antihistamines or leukotriene antagonists should be administered, as well as topical antihistamines or nasal glucocorticoids to treat allergy symptoms. However, there seems to be still room for improvement in the management of ARC patients because patients still report dissatisfaction with or do not find relief with commonly prescribed antihistamines, or glucocorticoids. This is partially due to the fact that available medications do not adequately reduce the signs and symptoms of rhinoconjunctivitis^2^, ^3^, ^4^ or because they cause unwanted side effects.^6^, ^8^, ^9^, ^31^ Generally, patients will not adhere if the efficacy of the therapy is not satisfactory. On the other hand, if reduction in symptoms is not sufficient, this may—at least partly—be due to nonadherence of patients to the treatment. This is also the case for unwanted side effects as their occurrence can also result in nonadherence to the treatment. This is a double‐edged issue: either the preparation does not work because it is not taken, or it does not work sufficiently, which is why it is not taken.

Because of these drawbacks, the interest in complementary and alternative medicine (CAM) for the treatment of ARC is rising. In an American landmark survey,^31^ 61% of the patients discontinued their prescribed treatment because of a characteristic of the treatment, rather than a change (positive or negative) of the symptoms, showing that the patients are not comfortable with the constituents of the treatment. A recent study showed that 40% of the American population uses CAM and 17% of the medications were used to treat otorhinolaryngologic diseases. This underlines the importance of alternative treatment approaches to cure rhinitis.^12^ A survey conducted by Schäfer et al.^13^ showed that 26.5% of patients used CAM for their allergies. Nevertheless, it is not possible to provide evidence‐based recommendations since the methodology used in most of the clinical trials with CAM for treatment of allergic rhinitis was frequently inadequate. Therefore, further studies are needed to investigate the effectiveness of CAMs in treating rhinitis.^10^ Natural products having a physical mode of action have already been approved by the FDA for the treatment of rhinitis.^32^ Other alternative products available to treat ocular symptoms include artificial tears, or liposomal eye sprays for rinsing allergens out of the eyes.^33^

The mode of action of ectoine in nasal and ophthalmic treatments is likely to be based on the physical interaction of ectoine with water and the resulting effects on the membranes of treated tissues.^18^ Potential mechanisms may include a stabilization of cell membranes, extracellular proteins, and other macromolecules to enhance the “barrier function” of tissues, reducing allergen–membrane interactions and thus inhibiting the initiation of the allergic cascade and subsequent inflammation.^34^ The functionality of the nasal and ocular epithelia is of crucial importance for the protection and as a barrier against invading allergens in rhinoconjunctivitis. Of note, the ectoine containing nasal spray and eye drops were developed as preservative free formulations, thus considering potential harmful side effects of preservatives such as the development of allergies or intolerances. Overall, only few side effects occurred that were related to the treatment. Of note, one AE, “burning eyes” was rated as unrelated by the investigator. It needs to be considered that it is quite likely that patients participating in the study presented here experienced allergic eye and/or nose symptoms because of airborne allergens, thus explaining the investigator's decision on the relationship between AE and treatment. Therefore, the investigator, who sees the patient on a regular basis during a study—and therefore can also best assess the AE—rated the AE described above as unrelated to the treatment.

The ability of ectoine to inhibit rhinoconjunctivitis signs and symptoms was demonstrated in the study presented here: treatment with ectoine containing nasal spray and eye drops provided clinically meaningful improvement in nasal, ocular and nonnasal signs and symptoms with a LSMD of ‒1.87 in the TNSS, ‒1.45 in the TOSS and up to a difference of ‒2.02 in the TNNSS.

The reduction in symptom scores following ectoine treatment is comparable with pharmacological therapies. This was shown in two noninterventional studies comparing the effectiveness of ENS and eye drops to azelastine eye drops and cromoglycic acid nasal spray.^25^ In the aforementioned study, the TNSS (as assessed by the physician) was reduced by 58.85% in the ectoine group, thus being similar to the 57.11% in the cromoglycic acid group. TOSS decreased by 45.96% in ectoine treated patients and by 44.98% in patients treated with cromoglycic acid nasal spray.^25^ This was also shown in a meta‐analysis comparing ectoine with different pharmacological treatments.^35^ These studies were all designed to treat currently present symptoms in contrast to the study presented here, applying a prophylactic approach. However, so far only a few noninterventional studies analyzed the effect of ectoine treatment. Therefore, further studies (with better evidence) are needed to substantiate the efficacy of ectoine.

Recently, Patel et al.^36^ conducted a study similar to the study presented here to test the effect of a combined olopatadine‐mometasone nasal spray (GSP301) and other treatments (olopatadine) in an EEC by analyzing the LSMDs of the different treatments. They showed that treatment with olopatadine improved instantaneous nasal symptoms in comparison to placebo with an LSMD of ‒0.81, which is much less than the LSMD for ectoine presented here. However, the other treatments tested in the study had all higher LSMDs (‒2.83 to ‒3.60). In contrast, ocular symptoms, as analyzed by instantaneous TOSS, were similar to the LSMD for ectoine, with LSMDs of ‒1.64 for twice‐daily treatment with GSP301 and ‒1.20 for once‐daily treatment with GSDP301. Hence, ectoine represents a useful protective element for the nasal and ocular epithelia to counteract the symptoms of ARC induced by pollen exposure in an EEC.

Salapatek et al.^37^ also conducted a study with mometasone furoate nasal spray as pre‐treatment before EEC exposure. The reduction of TNSS was similar to the reduction in the current study (data not shown), demonstrating that ectoine exerts equivalent effects in comparison to pharmacological treatment. The approach of treating eyes and nose simultaneously is based on the fact that over half of the allergy sufferers experience conjunctivitis symptoms in addition to their rhinitis symptoms when exposed to allergens.^38^, ^39^ This is due to two mechanisms: allergen introduced in the eyes drains with the tears on a direct way into the nose via the nasolacrimal duct and vice versa via indirect reflex connections from the nose to the eyes.^40^, ^41^ To overcome this cross‐linking, this study presented here was designed to test concomitant treatments for eyes and nose to assess the maximal clinical benefit.

Besides the ARC symptoms subjective assessment methods, AcR, which objectively assesses nasal patency and correlates with improvement of congestion is a valid, reproducible diagnostic method for measuring nasal cavity geometry that includes MCA.^37^ AcR is not influenced by subjective comparisons and thus more reliable in studies with relatively small number of patients. The MCA change in patients treated with ectoine was smaller (more than twofold) compared to patients treated with placebo, indicating a smaller decrease in nasal patency (i.e., smaller increase in congestion). Barchuk et al.^42^ showed that prophylactic treatment with a novel H_3_‐receptor antagonist resulted in a smaller decrease in MCA in comparison to placebo with a LSMD of ‒0.126. Though the effect of ectoine was not as pronounced as for the H_3_‐receptor antagonist, it has to be kept in mind that ectoine solely acts on the basis of physical interactions with water, in contrast to pharmacological treatments acting on histamine receptors.

This study has limitations: the use of placebo preparations also improved rhinoconjunctivitis symptoms by 12%. This effect of placebo solutions was also shown in other studies: the rinsing and/or clearing of allergens with saline solutions from the nose and eyes have been shown to provide temporary relief of symptoms.^43^ Importantly, the magnitude of the placebo effect seen in this study was at the lower end of the range compared to that seen in other studies of pharmaceuticals, which have reported placebo effects up to 40%.^44^ The EEC is an appropriate system to study allergic patients, since it provides a much more controlled environment than a real‐life situation. For example, it is difficult to assess the efficacy during a season with a poor pollen flight. However, harsher environmental situations/changes, like simultaneous exposure do different allergens, cannot be done in an EEC. Therefore, conducting studies in a real‐life setting would be an interesting approach to further analyze the efficacy of ectoine.

## 5 CONCLUSIONS

This study demonstrates that ectoine is effective in reducing mild to moderate rhinoconjunctivitis symptoms in a clinically relevant manner in seasonal ARC patients in an EEC. Thus, ENS and ectoine ophthalmic drops represent interesting nonpharmacological treatment options for ARC.

## CONFLICT OF INTERESTS

Anne M. Salapatek is an employee of Cliantha Research Limited a clinical, contract research organization. Nina Werkhäuser is an employee of Bitop AG, a company where medical devices, including the ectoine nasal spray and eye drops, are developed and registered. Basma Ismail is an employee of Cliantha Research Limited a clinical, contract research organization. Ralph Mösges reports personal fees from ALK, grants from ASIT Biotech, personal fees from allergopharma, personal fees from Allergy Therapeutics, grants and personal fees from Bencard, grants from Leti, grants, personal fees and nonfinancial support from Lofarma, nonfinancial support from Roxall, grants and personal fees from Stallergenes, grants from Optima, personal fees from Friulchem, personal fees from Hexal, personal fees from Servier, personal fees from Klosterfrau, nonfinancial support from Atmos, personal fees from Bayer, nonfinancial support from Bionorica, personal fees from FAES, personal fees from GSK, personal fees from MSD, personal fees from Johnson & Johnson, personal fees from Meda, personal fees and nonfinancial support from Novartis, nonfinancial support from Otonomy, personal fees from Stada, personal fees from UCB, nonfinancial support from Ferrero, grants from BitopAG, grants from Hulka, personal fees from Nuvo, grants from Ursapharm, outside the submitted work. Esther Raskopf was an employee of CRI—Clinical Research International Ltd., at the time of conducting the study and reports personal fees from Bayer and personal fees from Gesellschaft für Phytotherapie outside the submitted work. Andreas Bilstein is a former employee of Bitop AG and receives personal fees from Bitop AG.

## AUTHOR CONTRIBUTIONS

Andreas Bilstein and Anne M. Salapatek conceptualized the study. Anne M. Salapatek and organization performed the study. Andreas Bilstein and Anne M. Salapatek evaluated the data. Anne M. Salapatek's organization made the statistical analysis. Esther Raskopf prepared the figures. Andreas Bilstein, Anne M. Salapatek, Nina Werkhäuser, Basma Ismail, and Esther Raskopf wrote the manuscript. All authors approved the final version of the manuscript before submission.

## Supporting information

Supplementary Material
