# Supplementary material for: Effects of ectoine containing nasal spray and eye drops on symptoms of seasonal allergic rhinoconjunctivitis
Source: Clin Transl Allergy. 2021 Mar 24;11(1):e12006. doi: 10.1002/clt2.12006 (PMC8099269; doi:10.1002/clt2.12006)
Supplement: Supplementary file 1 — Supplementary Material [file CLT2-11-e12006-s001.pdf]

Supplemental File 1:

Nasal cavity geometry (minimal cross-sectional area) was measured using acoustic rhinometry (AcR). All measurements were completed by trained operators. As much as possible, the same equipment was used for each measurement on the same subject to ensure consistency during the study visit.

The absolute minimal cross-sectional area along the nasal passage 0 (at the nares) to 5.5 cm into the nasal passage were derived as follows:

Minimal cross-sectional area 1 (MCA 1) was captured within the nose at a distance of 0 and 2.2 cm. The minimal cross-sectional area 2 (MCA2) was captured within the nose at a distance of 2.2 and 5.5 cm. Distance was defined as the location of cross-section area measured in cm from the nares. MCA1 and MCA2 were captured simultaneously for each nostril. In all, 4 measurements were captured to determine the overall minimal MCA that occurred along the nasal passage in either nostril.

Three measurements were obtained from each nostril to demonstrate reproducibility and accuracy. These 3 measurements were collected such that there was ≤ 5% deviation between each round as indicated by the software in the measurement accuracy bar.

The 3 measurements from each nostril resulted in 12 data points:

3x MCA1 right

3x MCA left

3x MCA2 right

3x MCA2 left

To calculate the absolute minimum cross section area for all regions in the left or right nostril, the following calculation was performed:

- Using the 3 acceptable measurements, calculated the average for each of: left MCA1, right MCA, left MCA2, right MCA2
- Selected minimum value from these averages. This was the reportable MCA value.
